# Influence of risk behavior aggregation in different categories of physical activity on the occurrence of cardiovascular risk factors

**DOI:** 10.1186/1755-7682-6-26

**Published:** 2013-06-21

**Authors:** Aline Fernanda Barbosa Bernardo, Rômulo Araújo Fernandes, Anne Kastelianne França da Silva, Vitor Engrácia Valenti, Carlos Marcelo Pastre, Luiz Carlos Marques Vanderlei

**Affiliations:** 1 Faculdade de Ciências e Tecnologia. Programa de Pós-Graduação em Fisioterapia. UNESP, Universidade Estadual Paulista. R. Roberto Simonsen, 305.19060-900, Presidente Prudente, SP, Brazil

**Keywords:** Cardiovascular Disease, Risk Factors, Risk Behaviors, Physical Activity

## Abstract

**Background:**

We aimed to verify the association of risk behavior aggregation in different categories of physical activity (PA) with the presence of cardiovascular risk factors (RF) employees at a public university.

**Method:**

We analyzed data of 376 employees, which were visited in their workplace for measurement of weight, height and questionnaires to identify the risk behaviors and risk factors. Chi-square test was used to analyze the association between the dependent and independent variables and binary logistic regression was used to construct a multivariate model for the observed associations.

**Results:**

Associations were found between the aggregation of following risk behaviors: smoking, alcohol consumption and physical inactivity, considered in different categories of PA, and the increase in RF, except for the presence of hypertriglyceridemia. Individuals with two or more risk behaviors in occupational PA category are more likely to be hypertensive (3.04 times) and diabetes (3.44 times). For the free time PA category, these individuals were 3.18 times more likely to have hypercholesterolemia and for locomotion PA, more likely to be hypertensive (2.42 times) and obese (2.51 times).

**Conclusion:**

There are association between the aggregation of two or more risk behaviors and the presence of cardiovascular RF.

## Background

Adopting healthy lifestyle habits is related to the reduced presence of risk factors (RF) for the development of cardiovascular disease (CVD) [1,2]. In contrast, a higher number of RF and hence higher morbidity and mortality from CVD have been observed in individuals with unhealthy lifestyles [3-5]. Physical inactivity is associated with the presence of RF such as obesity [3], hypertension and increased risk for CVD [4]. Smoking is associated with high prevalence of dyslipidemias [6], higher frequency of alcohol consumption and reduced physical activity, and predisposition to the development of hypertension [7,8].

The effects of regular exercise on cardiovascular health are well documented [3,7,9-11]. On the other hand, although studies identified that physical activity (PA) of occupational nature (OPA) and free time (FTPA) may also result in cardiovascular protection [12,13]. The issue seems still little explored.

Searches in database indicated that scientific studies exploring the association of generalized physical activity (without distinction of domains) and isolated risk behaviors with RF are commonly found [3,7,9-13]. Moreover, studies analyzing the occurrence of RF in the aggregation of important risk behaviors (tobacco and alcohol) combined with a sedentary lifestyle in different fields of PA were not found. The distinction of the different fields of PA identifies which of these is more strongly associated with cardiovascular RF [12,13].

Therefore, we aimed to investigate the association of risk behavior aggregates in different domains of PA with the presence of cardiovascular RF in employees at a public university.

## Methods

### Study population

This is a cross-sectional epidemiological study in 376 subjects from the workforce of a large public university in São Paulo State, Brazil. This population was chosen because it has distinct characteristics between categories of PA. It was not included in the study those who refused to cooperate with the data collection and that were not found in the workplace after three visits. Taking into account these criteria we included 89.52% of the employees of the institution.

All volunteers were informed about the procedures and objectives of the study and, after agreeing they signed a consent form. All procedures used in the study were approved by the Ethics Committee in Research of the Institution (Proc. No. 96/2010) and complied with Resolution 196/96 of the National Health Council, 10/10/1996.

### Data collection

The employees were visited in the workplace by a properly trained staff. Initially, the volunteer was identified in relation to the name, age and sector work and we measured weight, height and blood pressure. Later, the volunteer replied, in individual interviews, questionnaires for identifying behaviors and risk factors.

### Risk behaviors

We evaluated three different health risk behaviors: smoking, alcohol consumption and physical inactivity. Smoking was considered positive when the subject consumed at least one cigarette per day. Alcoholism was nominated for respondents who reported daily consumption of any alcoholic beverage.

To assess physical inactivity we used the questionnaire developed by Baecke et al. [14], which was validated for the Brazilian population [15,16] and provides a score for the dimensionless current practice of PA. The questionnaire has as reference the last 12 months and assesses PA in distinct sections: 1) occupational physical activity (OPA), 2) free time physical activity (FTPA) and 3) locomotion physical activity (LPA) [14-16]. The classification of individuals as active or sedentary was made based on the score of each category of PA provided by the questionnaire. This classification took into account the criterion adopted by Codogno et al. [17] in which the value of each score was stratified into quartiles and were considered sedentary individuals located in the lowest quartile (P25).

The OPA was assessed taking into consideration the activities performed during occupational period, distinguishing the occupation type [18], the weather remained standing and the amount of weight carried by the individual while the working period [14-16]. The investigation of FTPA was done by assessing the practice of regular physical exercises involving specific modalities. Based on the intensity (light, moderate and vigorous) [18], for frequency and duration of activity it was calculated a score for that particular domain [14-16]. To evaluate the LPA, the questions referred to the activities of watching television (sedentary activity), walking, cycling, and one last question, regarding the total minutes per day on activities of locomotion (walk or use a bicycle to go to work, school or shopping) [14-16].

The risky behaviors were aggregated in each PA category and created a score ranging from zero (no risk behaviors) to three (presence of three behaviors [sedentary lifestyle, smoking and alcoholism]) risk behaviors from the number of behaviors risk that each individual reported.

### Risk factors

The risk factors evaluated were obesity, hypertension, hypercholesterolemia, hypertriglyceridemia and diabetes mellitus. Obesity was considered in individuals who present a body mass index (BMI) above 30 kg / m^2^[19]. To obtain the values of BMI, employees were instructed to be wearing light clothing that was held for the measurement of weight (kg) and height (m) through the balance Plenna Tin 00139 (Maximum, Brazil) and portable stadiometer Personal Caprice (Sanny, Brazil).

To evaluate the presence of hypertension, blood pressure was measured indirectly through aneroid sphygmomanometer (Welch Alyn - Tycos, New York, USA) and stethoscope (Littmann, Saint Paul, USA), with individuals seated following the recommendations of the VII Hypertension guidelines [20]. Values equal to or above 140/90 mmHg were considered positive for hypertension and volunteers who had values below these levels, but reported use of antihypertensive drugs or reported physician-diagnosed hypertension were also considered hypertensive.

Hypercholesterolemia, hypertriglyceridemia and diabetes mellitus were considered when the subject reported changes in the exams in the last year or use medication for that condition. Individuals who did not undergo tests in the last year or reported to be unaware of changes in examinations were not considered [7,11,21].

### Statistical analysis

For categorical variables we calculated: prevalence and confidence intervals (CI) of 95% (95%). The chi-square test analyzed the association between the dependent variable and the other independent variables. A binary logistic regression was used to construct a multivariate model for the observed associations. The independent variables that the chi-square test were associated with the dependent variable by 20% (p = 0.200) were entered simultaneously in the multivariate model. This process generated values adjusted odds ratio (OR) and their 95% CIs. Significance values (p-value) of less than 5% were considered statistically significant and all analyzes were performed in SPSS version 13.0 (Statistical Package for the Social Sciences Inc, Chicago, Illinois).

## Results

Table 1 presents the anthropometric characteristics of the study population and cardiovascular profile. In general, the average blood pressure for the group were within normal limits. On the other hand, there was high BMI, showing high prevalence of overweight in the sample.

**Table 1:** Mean values, followed by their standard deviation of anthropometric data and cardiovascular parameters of the study population

**Variables**	**Mean ± standard deviation**
Age (years)	44.37 ± 9.13
Weight (kg)	76.53 ± 16.7
Height (m)	1.67 ± 0.09
BMI (kg/m^2^)	27.04 ± 4.54
SAP (mmHg)	117.78 ± 15.12
DAP (mmHg)	79.11 ± 10.48

Legend: Kg = kilogram; m = meters; mmHg = millimeters of mercury; bpm = beats per minute; BMI – Body mass index; SAP = systolic arterial pressure; DAP = diastolic arterial pressure.

There was no association between risk behaviors and presence of hypertriglyceridemia in the different categories of PA (p-values ranging from 0.062 to 0.432). For OPA the association was found between risk behaviors and increased presence of hypercholesterolemia (p-value = 0.025), hypertension (p-value = 0.005), obesity (p-value = 0.007) and diabetes mellitus (p-value = 0.019). For the FTPA we found significant association between risk behaviors and hypercholesterolemia (p-value = 0.001), hypertension (p-value = 0.008) and diabetes mellitus (p-value = 0.030) and association of LPA with hypertension and risk behaviors pressure (p-value = 0.001) and obesity (p-value = 0.001) (Table 2).

**Table 2:** Association of risk behaviors with cardiovascular risk factors

		**Hypercholesterolemia**	**Hypertriglyceridemia**	**Hypertension**	**Obesity**	**Diabetes mellitus**
		%	%	%	%	%
Aggregate OPA	None	12.6	5.1	17.7	16.7	6.1
	Only 1 factor	16.0	9.7	22.9	27.3	7.6
	≥ 2 factors	29.4	11.8	41.2	32.4	20.6
	*p*	0.025	0.062	0.005	0.007	0.019
Aggregate FTPA	None	10.7	5.8	16.5	18.4	5.3
	Only 1 factor	18.7	10.4	27.6	27.1	10.4
	≥ 2 factors	30.6	5.6	30.6	25.0	13.9
	*p*	0.001	0.431	0.008	0.106	0.030
Aggregate LPA	None	13.8	6.6	14.9	16.0	6.1
	Only 1 factor	14.2	7.7	26.5	25.3	10.3
	≥ 2 factors	27.5	10.0	35.0	37.5	7.5
	*p*	0.098	0.432	0.001	0.001	0.356

Legend: OPA=occupational physical activity; FTPA=free time physical activity; LPA=locomotion physical activity.

Employees with two or more risk behaviors compared to those with no risk behavior in the OPA category were 3.03 times more likely to be hypertensive and 3.44 times more likely to be diabetic. For the FTPA category, subjects with two or more risk behaviors were 3.18 times more likely to have hypercholesterolemia and for the LPA category they were 2.42 times more likely to be hypertensive and 2.51 times to be obese (Table 3).

**Table 3:** Multivariate model to describe the association between behaviors and risk factors in adults

		**Hypercholesterolemia**	**Hypertension**	**Obesity**	**Diabetes mellitus**
		**OR§ (OR**_**95% CI**_**)**	**OR§ (OR**_**95% CI**_**)**	**OR§ (OR**_**95% CI**_**)**	**OR§ (OR**_**95% CI**_**)**
Aggregate OPA	None	1.00	1.00	1.00	1.00
	Only 1 factor	1.12 (0.59 – 2.12)	1.17 (0.67 -2.04)	1.54 (0.89 – 2.66)	1.25 (0.52 – 2.99)
	≥ 2 factors	2.18 (0.89 – 5.35)	3.03 (1.34 – 6.82)	2.24 (0.95 – 5.31)	3.44(1.21 – 9.77)
Aggregate FTPA	None	1.00	1.00	--	1.00
	Only 1 factor	1.62 (0.84 – 3.12)	1.63 (0.93 – 2.85)	--	1.80 (0.77 – 4.21)
	≥ 2 factors	3.18 (1.31 – 7.76)	1.88 (0.81 – 4.37)	--	2.38 (0.75 – 7.55)
Aggregate LPA	None	--	1.00	1.00	--
	Only 1 factor	--	1.80 (1.03 – 3.16)	1.59(0.90 – 2.79)	--
	≥ 2 factors	--	2.42 (1.09 – 5.38)	2.51 (1.14 – 5.54)	--

Legend: OPA=occupational physical activity; FTPA=free time physical activity; LPA=locomotion physical activity; OR = odds ratio; OR_IC95%_ = confidence interval of 95%; § = Model adjusted for age, sex and occupational group.

## Discussion

Although it is well described in the literature that unhealthy behaviors influence the presence of RF [4,22,23], little information exists on the aggregation of inactivity in different domains and other risk behaviors related to RF.

Smoking increases the heart post-workload by increased sympathetic activity and the effect of its chemical components contribute to greater availability of lipids in the bloodstream and subsequent accumulation of fat in the arteries [7]. The excessive consumption of alcohol may promote the elevation of blood pressure and triglyceride levels [23] and sedentary lifestyle predisposes to the development of obesity and arterial hypertension [2]. The background above makes clear the relevance of the RF aggregation to health, as well as the importance of studying different manifestations. Accordingly, among the analyzed risk behaviors, physical inactivity manifested in various fields seem to affect health in different ways.

In general, less active individuals at work that have incorporated other risk behaviors were more likely to present hypertension and diabetes mellitus. Classic studies conducted among British workers have reported the importance of physical activity in the prevention of occupational mortality from cardiovascular disorders [24]. Studies exploring isolated risk behaviors in the occupational domain described positive association of low levels of OPA with systolic and diastolic blood pressure and diabetes mellitus [25,26]. Likewise, it is known that although decreasing over the last years [27], tobacco and other legal drugs are more frequent among people with lower education level [28], which composed a great part of our sample. Furthermore, the use of legal drugs is frequent during tension relief [29], as experienced by employees of administrative sessions, which are less physically active at work. These results identify a focus of concern for managers involved in these health care employees. Individuals who are smokers, consume alcohol and perform light work activities should be encouraged to modify their risk behaviors and mainly perform regular PA [5,8].

In this study we observed the association of risk behaviors with hypertension, regardless of the PA category, however, for the FTPA category characteristics such as gender, age and operating group also seemed to influence this association. Primary prevention of hypertension is associated with three risk behaviors studied. Alcohol and tobacco promotes alterations on blood lipid components, and may change the blood pressure [7,23] and the sedentary habit is a behavior associated with hypertension [2,7,8]. These aggregate factors potentiate the occurrence of hypertension, which indicates the importance of these behaviors to the development of hypertension.

In this context, the sedentary habit added to other risk behaviors was associated with hypertension and obesity. Alcohol and tobacco promotes alterations in blood lipid components and are able to induce changes in blood pressure [7,23]. It is known that the leisure domain is the focus of various studies, since among all the domains it is the most vulnerable to be modified by campaigns promoting physical activity [24]. Previous studies showed that the practice of leisure activities throughout life may prevent obesity and hypertension [30] and that most active people in leisure have lower chances of consuming large amounts of alcohol [31], which may increase blood pressure and BMI [31]. These aggregates factors potentiate the occurrence of hypertension and obesity, which indicates the importance of these behaviors for the development of both outcomes.

As limitations of this study, we may raise the cross-sectional design, in which risk behaviors, risk factors and outcomes are observed at the same time, which may induce reverse causality bias. Moreover, identification of the presence of hypercholesterolemia, hypertriglyceridemia, and diabetes mellitus by questionnaire may also be considered a limitation, although it is used in studies of large populations [11,15-17,25,26].

Whereas the aforementioned characteristics may influence the chance of occurrence of RF in different categories of PA, further studies should be directed to understand the influence and magnitude that each feature has on the RF occurrence. Thus, studies that explore different subjects with occupational activities, leisure time and mobility and also different age and gender may clarify doubts and assist researchers and clinicians to develop strategies for lifestyle changes and, as a consequence, decreasing the development of CVD.

## Conclusion

In summary, our data demonstrate that, when aggregated, the risk behaviors increase the chance of the individual to present RF. Although this aggregation of risk behavior is harmful to the health domain regardless of PA, the inclusion of each of these areas in the aggregation is associated with different risk factors, thus, confirming the importance of considering them separately.

## Competing interests

The authors declare that they have no competing interests.

## Authors’ contributions

AFBB, RAF and AKFS participated in the acquisition of data. AFBB, RAF, AKFS, CMP, VEV and LCMV participated in the revision of the manuscript. All authors determined the design, interpreted the data and drafted the manuscript. All authors read and gave final approval for the version submitted for publication.
